# Histochemical Assessment of Reticulin–Collagen Patterns in the Mid-Secretory Endometrium Predicts Recurrent Pregnancy Loss

**DOI:** 10.3390/pathophysiology32020024

**Published:** 2025-06-03

**Authors:** Nazerke Oshakhtiyeva, Dmitriy Klyuyev, Zhanna Amirbekova, Rinat Gatin, Anar Turmukhambetova, Kamilya Makhambetova, Irina Kadyrova, Yevgeniy Kamyshanskiy

**Affiliations:** 1 Institute of Life Sciences, Karaganda Medical University, Karaganda 100008, Kazakhstan; oshahtieva_n@mail.ru (N.O.); turmukhambetova.a@amu.kz (A.T.); kamila-m09@mail.ru (K.M.); ikadyrova@qmu.kz (I.K.); 2 Department of Obstetrics and Gynecology, Karaganda Medical University, Karaganda 100008, Kazakhstan; amirbekovaz@kgmu.kz (Z.A.); frg.80@mail.ru (R.G.); 3 Clinic of Medical University, Karaganda Medical University, Karaganda 100000, Kazakhstan; kamyshanskiy@qmu.kz

**Keywords:** collagen, extracellular matrix, mid-secretory endometrium, histochemistry, histology, ultrasonography, clinical and biochemical pregnancy loss

## Abstract

Background/Objectives: Normal remodeling of the extracellular matrix of the endometrium is a necessary condition for the implantation of a blastocyst. We evaluated whether the use of histochemical reticulin–collagen staining can improve the assessment of the extracellular matrix of the mid-secretory endometrium in recrudescent clinical/biochemical pregnancy losses in comparison with ultrasound and routine histological examination. Methods: We compared the histochemical pattern of reticulin–collagen endometrial biopsy (21st day of the cycle) with ultrasonography and standard histological examination of the endometrium in the following groups: (1) fertile women with gravidity ≥ 2, (gravidity = parity) and (2) women with two or more clinical/biochemical pregnancy losses. Results: A normal pattern (NP) with ordered reticulin fibers forming cellular structures was determined in 92% of biopsies with physiological reproductive status and 44% of biopsies with recrudescent reproductive failure (*p* < 0.05), despite the fact that there were no differences in ultrasonography and standard histological examination between the groups (*p* > 0.05). A histochemical pattern of insufficient secretory endometrial transformation with abnormal noodle-like pattern (aNP) collagen fibers was more common in recrudescent reproductive failure (56%) than in women with physiological reproductive status (8%) (*p* < 0.001), despite the fact that insufficient secretory endometrial transformation with an abnormal noodle-like pattern with collagenization (aNPC) was detected only in recrudescent reproductive failure, and it was not detected in women with physiological reproductive status (*p* < 0.00001). Conclusions: We determined the histochemical pattern of the extracellular matrix of the endometrium in terms of the type of reticulin–collagen, associated in our study with recrudescent clinical/biochemical pregnancy loss, with improved predictability compared to ultrasonography and standard histological examination. We propose to use the method of histochemical evaluation of the reticulin–collagen pattern in order to stratify groups of women of fertile age at risk of reproductive failure.

## 1. Introduction

The endometrium is a unique tissue that plays an important role in reproduction. Its functional transformation throughout the menstrual cycle provides optimal conditions for embryo implantation. This process includes several critical stages: primary contact with the endometrium, introduction into the stroma, and maintenance of trophoblast invasion [1,2,3]. The successful completion of these stages depends on a strictly regulated endometrial microenvironment, in which the extracellular matrix acts as a key modulator. The endometrial extracellular matrix is a dynamic structure that forms a mechanical and biochemical environment for cellular adhesion, migration, and differentiation [4,5,6,7]. Among its components, collagen plays a special role, determining its biomechanical properties [8,9,10]. Collagen I forms strong fibrils that increase tissue rigidity, while reticulin is characterized by an increased elasticity and immaturity of its structure. The balance between these collagen types is important for the regulation of tissue stiffness and the stability of the extracellular matrix structure [11,12,13]. In addition, collagen affects a wide range of cellular processes, including cell migration and differentiation, as well as limiting excessive trophoblast invasion [14,15,16].

Scientific studies show that changes in collagen expression in the endometrium are associated with reproductive losses, including implantation failure [17,18,19,20]. These changes can also disrupt the process of the transformation of stromal fibroblasts into the decidual phenotype—a key stage of successful implantation [21,22,23]. Immunological, transcriptomic, and proteomic studies have shown that in miscarriage and infertility, the endometrium has structural and functional abnormalities compared to a normally functioning endometrium at the same stage of the menstrual cycle [24,25,26,27,28,29,30,31]. Furthermore, accumulating data indicate that changes in the endometrial microenvironment include an increased activity of matrix metalloproteinases, leading to excessive collagen degradation [24,32]. Such structural and molecular changes contribute to the maintenance and prolongation of adverse changes, which may lead to persistent changes in the extracellular matrix.

Despite significant advances in the study of the endometrial extracellular matrix, the identification of its microstructural histochemical features remains a challenge in routine clinical practice. Traditional diagnostic methods such as ultrasonography and standard histology have limited sensitivity for detecting microstructural changes in the extracellular matrix in reproductive dysfunction. The present study aims to fill one aspect of this gap. We describe the histochemical patterns of the extracellular matrix with an assessment of the distribution of reticulin and collagen in mid-secretory phase endometrial biopsies. By comparing the histochemical pattern of reticulin–collagen in women with physiological reproductive status and in women with recurrent reproductive losses, we present evidence for the utility of this diagnostic approach for stratifying women at risk of reproductive failure.

## 2. Materials and Methods

### 2.1. Study Design and Inclusion/Exclusion Criteria

Here, we present a blinded study conducted by comparing the histochemical pattern of the endometrium with ultrasound characteristics and the results of a standard histological examination of the endometrium in the middle stage of the secretion phase in groups of fertile women with physiological reproductive status and recrudescent reproductive failure. Patients were recruited between January and May 2024. Written informed consent was obtained from all patients before endometrial sampling. All methods were performed in accordance with the Declaration of Helsinki. This study was approved by the Local Bioethics Commission of the NCJSC “Karaganda Medical University” (Protocol No. 1 of 29 December 2023).

We formed two groups to study the histochemical pattern of the endometrium:

The group of women with physiological reproductive status (group 1) consisted of fertile women with at least two pregnancies with live fetus delivery in the anamnesis (the number of pregnancies was equal to the number of births).

The group of women with recrudescent reproductive failure (group 2) consisted of fertile women with at least two clinical or biochemical pregnancy losses or at least two unsuccessful cycles of extracorporeal fertilization with embryos of good quality.

Clinical data were obtained from medical records in a complex medical information system.

Exclusion criteria: (1) women under 20 years of age or over 40 years of age, body mass index (BMI) > 29.9 kg/m^2^; (2) congenital uterine abnormalities or acquired uterine diseases, including endometrial polyps, submucous myomas, uterine synechiae, and adenomyosis; (3) chronic endometritis; (4) hydrosalpinx; (5) positive antibodies to lupus anticoagulant and anticardiolipin; (6) gynecological operations in the preceding two months, or intrauterine device or any form of hormonal contraception during the six months preceding the study; (7) genetic abnormalities of the fetus; (8) elevated or decreased levels of thyroid stimulating hormone (TSH), follicle-stimulating hormone (FSH), luteinizing hormone (LH), or estradiol (E2); (9) polycystic ovary syndrome.

**Figure 1:**
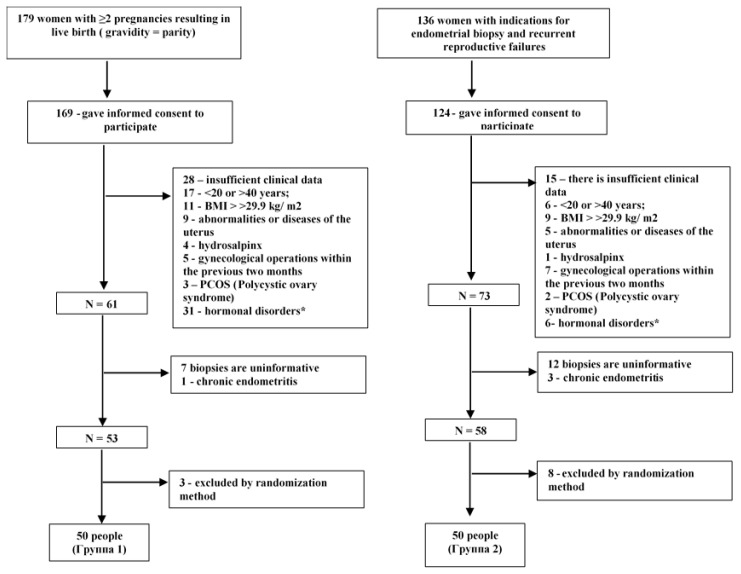
Study design. Recruitment and classification of women into two groups for endometrial biopsy analysis. Abbreviations: BMI—body mass index; PCOS— polycystic ovary syndrome. Notes: * hormonal disorders imply an increased or decreased level of thyroid-stimulating hormone, follicle-stimulating hormone, luteinizing hormone, or estradiol. Definitions of groups: Group 1 is a group of women with physiological reproductive status; Group 2 is a group of women with recurrent reproductive disorders.

The design of this study is shown in Figure 1.

In our study, physiological reproductive status was defined as the ability to achieve clinical pregnancy without assisted reproductive technologies and with a live fetus delivery.

In our study, reproductive failure was defined as the absence of pregnancies (infertility type 1 or infertility type 2) or the spontaneous termination of clinical pregnancy (loss of pregnancy after spontaneous conception, assisted reproductive technologies, ectopic pregnancy, hydatidiform mole) or biochemical pregnancy (implantation failure).

Biochemical pregnancy is a pregnancy diagnosed based on the following set of signs: (1) β-HCG (<100 mlU/mL); (2) a rapid drop in the concentration of β-HCG in urine or serum [33].

Clinical pregnancy is a pregnancy diagnosed by ultrasound imaging of one or more intrauterine embryos or obvious clinical signs of intrauterine pregnancy [33].

Stimulated cycles (SCs) are a pharmacological therapy in women to form regular ovulatory cycles [34].

Natural cycles (NCs) are menstrual cycles without pharmacological drug usage [34].

Assisted reproductive technologies involve the in vitro processing of human oocytes and spermatozoa or embryos for reproduction [33].

### 2.2. Sample Size Calculation

The formula for comparing two independent proportions was used to calculate the sample size, assuming a significance level of 5% and a test power of 80%. The initial proportions for the two groups were p1 = 0.70 (physiological reproductive status group) and p2 = 0.40 (recurrent reproductive loss group), based on the results of the preliminary analysis of our pilot study, which resulted in an estimated sample size of 40 participants in each group. To account for possible dropouts or exclusions, the sample was increased by 20%, after which the result was rounded to the nearest ten. As a result, the total sample size was 100 participants, equally distributed between the two groups.

The sample size calculation was performed using standard statistical methods [35] and verified using G*Power software, version 3.1 (Heinrich-Heine-Universität Düsseldorf, Germany) [36].

### 2.3. Methodology of Ultrasound Assessment of the Endometrium

A General Electric Voluson E8 ultrasound system with a 2D vaginal sensor with 5–8 MHz frequency was used for ultrasonography examination. All ultrasonography studies were conducted by the same doctor using the same ultrasound device.

Transvaginal ultrasonography was performed in the morning before the endometrial biopsy. The estimated parameters included the thickness of the endometrium [37,38,39,40] and the homogeneity/heterogeneity of the echo of the functional layer of the endometrium [37,38,40,41] with the presence/absence of a central echogenic line of the endometrium [42] according to the approved protocol.

The thickness of the endometrium was evaluated as the distance between the border of the endometrium and the myometrium of the anterior and posterior walls of the uterus, measured at a distance of 2 cm from the uterine fundus in the projection of the median longitudinal axis of the uterus. The measurement was carried out three times, and the average value was calculated. Images of the endometrium in the longitudinal direction were taken on the 21st day of the cycle. Clinicians examined the pattern and thickness of the endometrium on the day of the examination. After that, two researchers re-evaluated the endometrial echo simultaneously to assess the homogeneity and visibility of the median line.

An echogenic endometrium [43] with a thickness of more than 7 mm [44,45,46] was regarded as the ultrasound pattern corresponding to the endometrium in the secretion phase.

### 2.4. Methods of Endometrial Biopsy Sampling and Their Histological Examination

All endometrial biopsies were performed on an outpatient basis using an aspiration curette (Pipelle de Cornier, Prodimed, Neuilly-en-Thelle, France). Patients with natural cycles were monitored daily via transvaginal ultrasonography from cycle day 10 to 12, continuing until the dominant follicle reached a diameter of 16 mm and subsequently disappeared. Simultaneously, urinary luteinizing hormone (LH) concentrations were assessed daily using commercial test kits. The day of follicular disappearance on ultrasound was designated as the day of ovulation.

Endometrial biopsies were scheduled individually: in natural cycles, the biopsy was performed 7 days after the urinary LH surge; in stimulated cycles, the biopsy was conducted on the 7th day following the initiation of progesterone therapy, corresponding to day 21 of the menstrual cycle.

Biopsies were taken from the fundus and upper part of the anterior and posterior walls of the uterus to assess the zones of maximum physiological development [47].

After fixation with 10% formalin at 4 °C for 24 h, sections with a thickness of 5 microns were stained with hematoxylin and eosin (H&E), Masson trichrome [48], and Gomori’s silver plating [49] at room temperature according to standard protocols.

Morphometric analysis of histological preparations was carried out blindly by two independent pathologists. In case of disagreement, a consensus diagnosis was made.

The main morphometric measurements and photographs were carried out using a light microscope and digital color microphotography with «Image» software. Material with an area of more than 1 cm^2^ was considered a sufficient volume for this study.

Histological examination was performed according to a standard established protocol (Mayer’s hematoxylin, Bio-Optica (Milano, Italy)).

#### 2.4.1. Glandular Epithelium

The pattern of the glandular epithelium of the endometrium was evaluated using established criteria [50,51]. When dating the endometrium, the days of the menstrual cycle and histological dating criteria were considered [50,52]. The histological diagnosis of the glandular epithelium pattern mismatch in the middle stage of secretion was defined as the asynchronous development of the glands with a delay/advance of the cycle day of more than 3 days [53].

#### 2.4.2. Pinopodes

Pinopodes are a cytoplasmic evagination of the apical surface of the cytosolic membrane of the glandular epithelium of the endometrium [54,55,56,57].

According to the relative number of pinopodes, the micropreparations were divided into two subgroups [58]:

‑ numerous, densely packed microvilli occupying most (>50%) of the apical membrane of epithelial cells.
‑ few rare microvilli occupying less than half (<50%) of the apical membrane of epithelial cells

#### 2.4.3. Reticulin Fibers of the Extracellular Matrix

The histochemical examination was carried out by Gomori’s silver plating (a kit of reticulum stains (modified Gomori’s) (ab236473), Bio-Optica (Milano, Italy)) according to the standard research protocol. Reticulin fibers were defined as black or dark brown fibers with gray nuclei.

#### 2.4.4. Collagen Fibers of the Extracellular Matrix

The histochemical examination was carried out by staining (Trichrome Stain Kit (Connective Tissue Stain) (ab150686), Bio-Optica (Milano, Italy)) according to the standard research protocol. Collagen fibers were defined as dark blue fibers with black nuclei.

#### 2.4.5. Interpretation of the Histochemical Pattern of the Extracellular Matrix of the Endometrium by Reticulin–Collagen Phenotype

The histochemical pattern was determined under a light microscope at ×100 magnification. 

The typical normal pattern (NP) of endometrial extracellular matrix remodeling in the middle stage of the secretion phase according to reticulin–collagen type was defined as reticulin fibers arranged in an orderly manner, forming clear cellular structures, and thin, filamentous, ordered collagen fibers, located mainly around blood vessels and glands. This pattern prevails in over 70% of the histological section.

Abnormal remodeling of the endometrial extracellular matrix in the middle stage of the secretion phase according to reticulin–collagen type has two phenotypes: (1) abnormal noodle-like pattern without collagenization (aNP) and (2) abnormal noodle-like pattern with collagenization.

The abnormal noodle-like pattern without collagenization (aNP), or an insufficient secretory endometrial transformation, is defined as a pattern in which reticulin fibers take on a wavy, tortuous shape, resembling “boiled noodles”. These fibers have a chaotically intertwined, wavy appearance, forming loops, turns, and twists with areas of rarefaction and disorganization. Importantly, collagen I fibers remain thin, filiform, and uniformly distributed in the endometrial stroma around blood vessels and glands, without evidence of focal or diffuse deposits of dense homogeneous collagen masses. This reticulin pattern must occupy more than 30% of the histological section to meet the diagnostic criteria for aNP.

The abnormal noodle-like pattern with collagenization (aNPC), or an insufficient secretory endometrial transformation with collagenization, is characterized by a combination of changes in reticulin structure (noodle-like pattern) and clusters of collagen fibers. A distinctive feature is the presence of focal or diffuse dense deposits of homogeneous masses of collagen I in the endometrial stroma. These changes occupy more than 30% of the area of the histological section and reflect significant changes in the structure of the extracellular matrix.

The patterns are determined under magnification ×100 for a general overview and ×400 for a detailed assessment of the morphological features of reticulin and collagen fibers.

### 2.5. Statistical Analysis

The obtained data were subjected to statistical processing using parametric and nonparametric analysis methods. The analysis, systematization, and visualization of the obtained results were carried out using the IBMSPSS Statistics v.22 program (StatSoft, Inc., Tulsa, OK, USA). Quantitative variables were initially analyzed using the Shapiro–Wilk test to determine the normality of distribution using the Levene test to check the homogeneity of variances. For quantitative characteristics, if the distribution was recognized as normal, the mean, standard deviation, and 95% confidence interval (95% CI) were calculated. Sets of quantitative indicators, with a different distribution, were described using the median (Me) and the upper and lower quartiles (Q1–Q3). Nominal data are presented as absolute values and percentages. To compare the frequencies of distribution by qualitative characteristics between groups, the chi-square statistical test with Yates’ correction or Fisher’s exact test was used. Proportions are presented with 95% confidence intervals (CI) calculated by the Clopper–Pearson method. To compare independent populations in cases where there were no signs of normal data distribution, the Mann–Whitney U test was used. When comparing mean values in normally distributed populations of quantitative data, the independent Student’s *t*-test was calculated. Values were considered statistically significant at *p* < 0.05.

## 3. Results

### 3.1. Demographic and Clinical Characteristics of the Study Groups

The demographic and baseline clinical characteristics of women from groups with physiological reproductive status and recrudescent reproductive failure are presented in Table 1.

The groups were comparable in age, BMI, and cycle duration and did not differ in serum levels of TSH, FSH, LH, and E2 measured on the 2nd or 3rd day of the menstrual cycle. At the same time, women with recrudescent reproductive failure had a more significant number of clinical and biochemical pregnancies in the anamnesis and, in 34 (68%) cases, had stimulated cycles.

### 3.2. Characterization of Histochemical Patterns of Reticulin and Collagen in the Endometrial Extracellular Matrix

**Figure 2:**
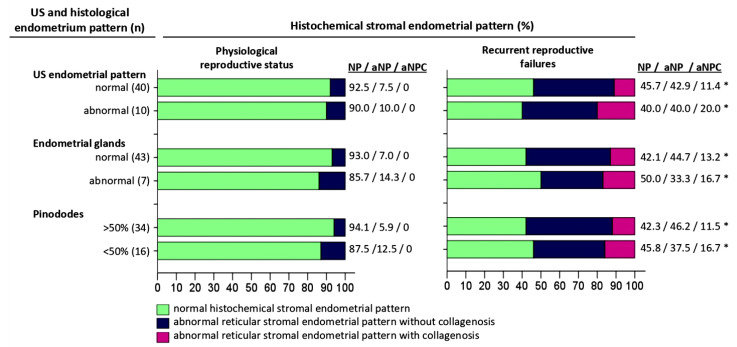
Comparative characteristics of histochemical pattern, ultrasound, and histological pattern of the endometrium from the middle stage secretion phase of menstrual cycle. Abbreviations: US—ultrasonographic investigation; NP—normal histochemical reticulin–collagen stromal endometrial pattern; aNP—abnormal reticular stromal endometrial pattern without collagenization; aNPC—abnormal reticular stromal endometrial pattern with collagenization. Notes: * Statistically significant difference versus physiological reproductive status (*p* < 0.05).

**Figure 3:**
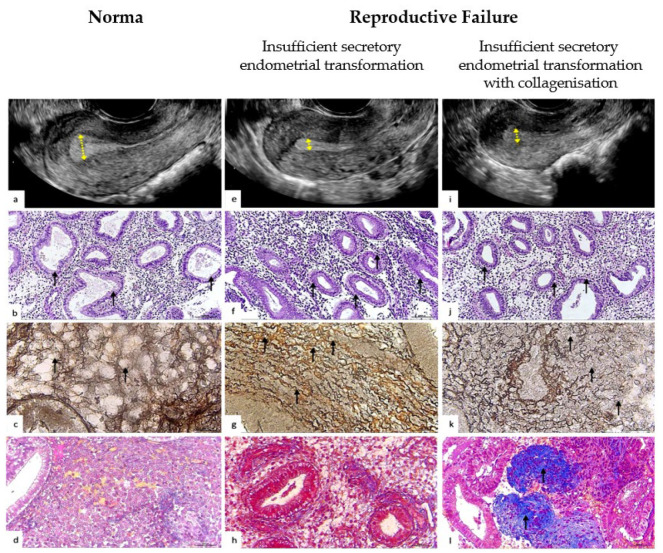
Representative ultrasonographic, histological, and histochemical endometrium patterns for physiological and pathological reproductive status. (**a**–**d**) Ultrasound and histopathological pattern of the endometrium with normal secretory transformation in the middle stage of the secretory phase ovarian cycle. (**a**) Ultrasonography. Homogeneous echogenic endometrium, endometrial thickness—12 mm (arrows). The echogenic pattern is typical for the secretory phase of the menstrual cycle. (**b**) Histopathological pattern. Convoluted endometrial glands with pseudostratified nuclei and a vacuolated cytoplasm, distinct subnuclear vacuoles located predominantly apically (arrows). The pattern of the glandular epithelium corresponds to the middle stage of the secretion phase. H&E staining: ×100. (**c**) Histochemical pattern of stromal reticular fibers (collagen type III). Reticulin fibers are arranged in an orderly manner, forming clear cellular structures; compartmented reticular stromal structure with formation of endometrial stromal channels (arrows). Gomori’s staining: ×400. (**d**) Histochemical pattern of stromal collagen (collagen type I). Thin filamentous collagen fibers located in the stroma of the endometrium (arrows). Masson’s trichrome staining: ×100. (**e**–**h**) Ultrasound and histopathological pattern of the endometrium with insufficient secretory endometrial transformation: abnormal noodle-like pattern collagen fibers (aNP). (**e**) Ultrasonography. Thin endometrium, thickness 5.5 mm (arrows). (**f**) The pattern of the glandular epithelium with delayed secretory transformation corresponds to the early stage of the secretion phase. Endometrial glands with pseudostratified nuclei and vacuolated cytoplasm and distinct parabasal vacuoles (arrows) H&E staining: ×100. (**g**) Histochemical pattern of stromal reticular fibers (collagen type III). Reticulin fibers are disordered, with a wave-like, tortuous, “noodle-like” pattern (arrows). (**h**) Normal, fine-thread-like, ordered collagen fibers, located mainly around the vessels and glands. (**i**–**l**) Ultrasound and histopathological pattern of the endometrium with insufficient secretory endometrial transformation with collagenization (aNPC). (**i**) Ultrasonography. Thin endometrium, thickness 6.5 mm (arrows). (**j**) Histopathological pattern. Endometrial glands with pseudostratified nuclei and vacuolated cytoplasm and distinct parabasal vacuoles (arrows). H&E staining: ×100. (**k**) Reticulin fibers with diffuse edema with microfragmentation and areas of disorganization (arrows). Gomori’s staining: ×400. (**l**) Histochemical pattern of stromal collagen (collagen type I). Abnormal foci of periglandular and perivascular deposition of homogeneous collagenous masses (arrows). Masson’s trichrome staining: ×100.

The histochemical pattern of endometrial extracellular matrix remodeling according to reticulin–collagen type is demonstrated in Table 2 and Figure 2 and Figure 3.

Among 46 (92%) (95% Cl:82.2–97.1%) endometrial biopsies of women with physiological reproductive status, NP was determined in 4 (8%) (95% Cl:3.0–17.9%) aNP biopsies; there were no cases with aNPS in this group.

In the group of women with recrudescent reproductive failure, NP was detected in 22 (44%) (95% Cl:30.6–58.2%) biopsies, of which 7 (28.6%) had NCs and 15 (71.4%) had SCs. aNP was observed in 21 (42%) (95% Cl:28.2–55.9%) biopsies, of which 6 (28.6%) had NCs and 15 (71.4%) had SCs. aNPC was detected in 7 (14%) biopsies, of which 3 (42.9%) had NCs and with 4 (57.1%) had SCs.

### 3.3. Comparative Analysis of Histochemical Patterns of the Endometrial Extracellular Matrix and Endometrial Patterns on Ultrasonography

The ultrasonography patterns of the endometrium are presented in Table 3 and Figure 3.

In the group with physiological reproductive status, in 40 (80%) (95% Cl:73.7–88.9%) women, a homogeneous endometrium with a central echogenic line and a thickness of more than 7 mm was determined by ultrasonography examination (Figure 2); of these, 37 (92.5%) endometrial biopsies had NP and 3 (7.5%) had aNP. On the other hand, the ultrasonography pattern did not correspond to the stage of secretion in 10 (20%) (95% Cl:10.7–32.4%) biopsies, of which 9 (90%) were NP cases and 1 (10%) was a case of aNP.

In the group of women with recrudescent reproductive failure, an ultrasonography pattern corresponding to the middle stage of the secretion phase was observed in 35 (70%) (95% Cl:58.9–78.3%) cases, among which there were 16 (45.7%) NP, 15 (42.9%) aNP, and 4 (11.4%) aNPC biopsies; 10 (28.6%) cases had NCs and 25 (71.4%) had SCs. On the other hand, an ultrasonography pattern not corresponding to the middle stage of the secretion phase was detected in 15 (30%) (95% Cl:19.7–43.9%) biopsies, among which 6 (40%) were NP, 6 (40%) aNP, and 3 (20%) aNPC; 6 (40%) had NCs and 9 (60%) SCs.

### 3.4. Comparative Analysis of Extracellular Matrix Histochemical Patterns and Endometrial Glandular Epithelium Histological Features

The histophenotype of the endometrial extracellular matrix in the middle stage of the secretion phase and the histological pattern of the glandular endometrium are presented in Table 4 and Figure 3b,f,j.

**Figure 4:**
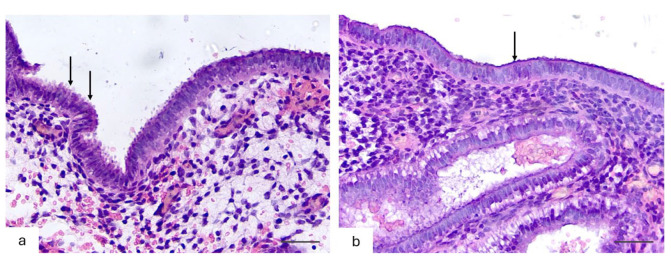
Histological pattern of the endometrial surface epithelium in the middle phase of secretory transformation (21 days) in women with physiological reproductive status and recurrent reproductive failure/loss (H&E staining, ×400). (**a**) Endometrium of a woman with physiological reproductive status. Numerous pinopodes—apical protrusions of the cytoplasm, occupying more than 70% of the epithelial cell surface (indicated by arrows)— are clearly visible on the epithelial surface. (**b**) Endometrium of a patient with recurrent reproductive loss/failure. Apical protrusions are present singly and occupy less than 20% of the epithelial surface (indicated by arrow).

In the group of women with physiological reproductive status, 43 (86%) (95% Cl:78.9–92.9%) biopsies showed convoluted endometrial glands with pseudo laminated nuclei and a vacuolized cytoplasm, with clear subnuclear vacuoles located mainly apically and with the presence of secretions in the lumen of the glands (Figure 4); of these, 40 (93%) were NP and 3 (7%) were aNP endometrial biopsies. The histological pattern of the glandular epithelium did not correspond to the middle phase of the secretion stage in 7 (14%) (95% Cl:6.2–26.1%) biopsies, of which 6 (85.7%) were NP and 1 (14.3%) was aNP.

In the group of women with recrudescent reproductive failure, in 38 (76%) (95% Cl:64.4–84.2%) cases, the histophenotype of the endometrial glands corresponded to the middle stage of the secretion phase; of these, 16 (42.1%) were NP, 17 (44.7%) were aNP, and 5 (13.2%) were aNPC biopsies, 11 (28.9%) with NCs and 27 (71.1%) with SCs. The histological pattern of the glands did not correspond to the intermediate stage of the secretion phase in 12 (24%) (95% Cl:13.9–37.4%) biopsies, of which 6 (50%) were NP, 4 (33.3%) were aNP, and 2 (16.7%) aNPC; 5 (41.7%) biopsies had NCs and 7 (58.3%) had SCs.

### 3.5. Comparative Analysis of Histochemical Patterns of the Extracellular Matrix and the Number of Pinopodes in the Endometrial Epithelium in the Middle of the Secretory Phase

The relative numbers of pinopodes *of the superficial endometrial epithelium* are presented in Table 5 and Figure 4.

In the group of women with physiological reproductive status, pinopodes covering most (>50%) of the apical cell membrane were observed in 34 (68%) (95% Cl:57.6–77.6%) biopsies, of which 32 (94.1%) had NP and 2 (5.9%) had aNP. Pinopodes covering a minor part (<50%) of the apical cell membrane were detected in 16 (32%) (95% Cl:20.9–43.8%) biopsies, of which 14 (87.5%) were NP and 2 (12.5%) were aNP cases.

In the group of women with recrudescent reproductive failure, pinopodes covering >50% of the apical cell membrane were observed in 26 (52%) (95% Cl:40.0–63.5%) biopsies, of which 11 (42.3%) had NP, 12 (46.2%) had aNP, and 3 (11.5%) had aNPC; 9 (34.6%) biopsies had NCs and 17 (65.4%) biopsies had SCs. On the other hand, pinopodes covering less than 50% of the apical membrane were detected in 24 (48%) (95% Cl:36.5–60.0%) biopsies, of which 11 (45.8%) had NP, 9 (37.5%) had aNP, and 4 (16.7%) had aNPC; 7 (29.2%) were NC and 17 (70.8%) were SC cases.

### 3.6. Comparative Analysis of Reproductive Outcomes in a Group of Women with Recurrent Reproductive Failure

During the dynamic observation of patients after primary endometrial diagnosis with histophenotyping of the extracellular matrix and subsequent comprehensive treatment (including immunomodulatory, angioprotective, and hormonal therapies), positive results were obtained.

Following the therapy, 22 clinical pregnancies (44%) with favorable perinatal outcomes were observed in the group of women with recurrent reproductive failure. Of these, 10 pregnancies (45.5%) were noted in patients in whom signs of incomplete/pathological remodeling of reticulin and collagen fibers were identified during the initial histological examination.

## 4. Discussion

We compared the histochemical pattern of the middle secretory endometrium with the ultrasonography pattern, the histological pattern of the glandular epithelium, and the relative number of pinopodes between groups of women with recrudescent reproductive failure and physiological reproductive status.

We found that the histochemical pattern of reticulin–collagen type in the endometrial extracellular matrix differs in women with physiological reproductive status and with recrudescent reproductive failure (*p* < 0.05). Thus, we identified two histochemical patterns. The first pattern had ordered reticulin fibers forming clear cellular structures (Figure 3c), associated with physiological reproductive status in more than 90% of cases.

The second type of extracellular matrix is characterized by disordered reticulin fibers that acquire a wavy, tortuous shape, forming loops, turns, and twists resembling “boiled noodles” (Figure 3g), with diffuse rarefactions and areas of disorganization (Figure 3k). This type was observed in more than 50% of cases of recurrent reproductive disorders.

This stromal pattern, which is often associated with reproductive failure, has two main phenotypes: (1) an abnormal noodle-like pattern without collagenization (aNP) (Figure 3h) and (2) an abnormal noodle-like pattern with collagenization (aNPC) (Figure 3l). We believe that the abnormal histochemical phenotype of reticulin–collagen of the endometrial stroma may reflect a pathological remodeling of the endometrial extracellular matrix in the middle stage of the secretion phase.

Further, we found that the abnormal reticulin–collagen pattern of the endometrial stroma was detected in all groups, in less than 10 percent of cases with physiological reproductive status and in more than 50% of cases with recrudescent reproductive failure, despite the fact that pathological remodeling of the extracellular matrix with collagenization, characterized by focal and diffuse easily visually detectable collagen bundles, was noted only in cases of recrudescent reproductive failure: no cases of collagenization were found in patients with physiological pregnancy. Qualitative and quantitative disorders and collagen abnormalities in the stroma were previously described in the intestine with collagenous microscopic colitis [59,60,61] and decidual tissue with clinically unexplained recurrent pregnancy loss [20,62]. It is unclear whether abnormal collagen expression is the cause or result of recrudescent miscarriage, but as previously shown [63,64,65,66], collagen may play an important role in indicating immune tolerance. Furthermore, an adequate remodeling of the collagenous extracellular matrix of the endometrium of the middle phase of the cycle plays an important role in forming a favorable implantation field and the diffusion and perfusion potential of the endometrium for implanted blastocytes. Our findings are consistent with and complement previous studies [30,67] by showing that focal collagen remodeling plays an important role in recurrent reproductive failure. This observation highlights the need for further studies to refine our understanding of the dynamics of interactions between structural components of the endometrial extracellular matrix in the context of reproductive failure.

Further, we showed that in the group with physiological reproductive status, in more than 90% of biopsies, clearly formed mesh-like cellular structures of the «honeycomb» type with evenly distributed thin reticulin fibers and single filamentous collagen fibers were determined. Similar structures were described earlier in chorionic villi, represented as stromal channels, the value of which is defined as the formation of connecting links with the function of transport and exchange between the mother and fetus [68]. We believe that a normal remodeling of proteins, in particular reticulin–collagen, in the extracellular matrix of the endometrium with physiological reproductive status is necessary for the formation of stromal channels to increase and improve the diffusion potential of the endometrium during the implantation of blastocysts. Previously, it was shown that pronounced edema develops in the endometrial stroma during implantation [69]. In our study, in more than 50% of cases with recrudescent reproductive failure, we found histopathological signs of diffuse edema of the endometrial stroma with a fibrinization and disorganization of reticulin and collagen fibers. In contrast, in the group with physiological reproductive status, such a histopattern was absent and the samples were characterized only by the stroma’s micro-focal rarefaction. We believe that the normal remodeling of the endometrial matrix is associated not so much with stroma edema as with the formation of a highly specialized stromal skeleton with the presence of stromal channels, and stromal edema is highly likely to be a pathological structural change in the endometrium which can mimic the normal ultrasound and histological pattern.

We found that endometrial ultrasonography patterns and histophenotypes differed between the groups. In the group with recrudescent reproductive failure, cases were often associated with a thin endometrium (Figure 3e,i,f,j), delayed maturation of the glandular epithelium, and a low number of pinopodes in comparison with the endometrium with physiological reproductive status.

Cases with stimulated menstrual cycle and reproductive failure were more often associated with ultrasonographic and histological signs of a mature and complete endometrium, in comparison with the subgroup with natural menstrual cycle and reproductive failure. This finding confirms previously published data that hormonal stimulation of the cycle accelerates the endometrium’s maturation and increases the endometrium’s thickness [70,71]. However, statistically significant differences were not found between the hormonally induced and non-induced cycles (*p* > 0.05) when assessing the histochemical phenotype of reticulin–collagen. This may indicate the existence of endogenous factors that play a key role in the pathological regulation of the endometrial extracellular matrix, not directly related to hormonal stimulation, which are manifested in both induced and natural cycles and require further study.

Our study showed significant differences in the histopatterns of the endometrial extracellular matrix in patients with physiological reproductive status and recurrent reproductive failure. However, it remains unclear whether these changes are the cause of reproductive disorders or their consequence.

Persistent inflammation and hormonal imbalance can initiate structural disorders of the extracellular matrix, creating unfavorable conditions for blastocyst implantation. In particular, proinflammatory cytokines mediate the hyperactivity of matrix metalloproteinases, which leads to a degradation of key components of the extracellular matrix and a disorganization of its structure [72,73,74]. Genetic factors, particularly mutations in genes encoding matrix components such as collagen or matrix metalloproteinases, may also reduce the ability of the endometrium to support blastocyst implantation and trophoblast invasion. On the other hand, implantation failure may trigger compensatory remodeling characterized by excessive collagen accumulation and dysfunctional matrix regulation. This may form a “vicious circle” where abnormalities in the structure of the endometrial extracellular matrix enhance subsequent reproductive failure. Longitudinal studies aimed at studying the dynamics of the histophenotype of the endometrial extracellular matrix at different periods of the menstrual cycle, as well as at different stages of pregnancy planning, are needed to elucidate this relationship in more detail.

We also identified two (4%) cases with a thin and immature histological pattern and an abnormal histochemical endometrial pattern in the group with physiological reproductive status; these were associated with unfavorable pregnancy outcomes and with placental abruption and intrauterine fetal growth retardation. Previously, several papers showed that a pathologically thin and immature endometrium for the phase of menstrual cycle is often associated with antenatal hypoxic–ischemic events in the placenta and fetus [75,76]. Therefore, we believe that pathological remodeling of the extracellular matrix of the endometrium is a high-risk factor not only for reproductive failure but also for long-term adverse consequences of pregnancy. However, this statement is speculative and requires further study.

It can be assumed, on the one hand, that the discrepancy between endometrial ultrasonography and histopatterns may be associated with a delay in the growth and maturation of the endometrium; on the other hand, it may be due to the persistence of insufficient endometrial remodeling, cases of which may be indirectly indicated by the collagenization of the extracellular matrix in the group with recrudescent reproductive losses; in our study, collagenization was not detected in patients with physiological reproductive status. We propose the use of the term «insufficient secretory endometrial transformation», which refers to the structural inconsistency of the glandular or extracellular matrix of the endometrium for the phase of the menstrual cycle according to a combination of ultrasonography, histological, and histochemical examination methods of the endometrium and which allows us to stratify a group of women at high risk of insufficient/abnormal formation of the endometrial implantation platform with a structural inconsistence of the epithelial–stromal compartment of the endometrium.

We hypothesize that such pathological remodeling of the extracellular matrix may be associated with immunopathological processes, as dynamic observation showed a positive effect following diagnostic and therapeutic interventions, including immunomodulatory and angioprotective therapies, as well as hormonal profile correction, which resulted in improved reproductive outcomes in patients with insufficient secretory endometrial transformation.

The strengths of this study include comparative characteristics of the histophenotypic pattern of the endometrium in the mid-secretory phase in recrudescent reproductive failure and in physiological reproductive status. The limitations of this study include data fluctuations, which may be due to the small number of cases in subgroups, as well as the lack of long-term follow-up to assess the impact of the identified histophenotypes on conception and pregnancy outcomes. Conducting multicenter studies with an increased sample size and taking into account different cycle stimulation protocols will increase the representativeness and validity of the results. To clarify the prognostic role of histochemical patterns of the endometrial extracellular matrix, it is necessary to conduct long-term longitudinal studies that will assess the impact of histophenotypes on conception, implantation success, and pregnancy outcomes. These studies will contribute to the development of a personalized approach in reproductive medicine, clarify the prognostic significance of the identified histophenotypes, and facilitate their integration into clinical practice to improve the risk stratification and prognosis of reproductive losses.

## 5. Conclusions

We determined the histochemical pattern of the extracellular matrix of the endometrium by the type of reticulin–collagen, associated in our study with recrudescent clinical/biochemical pregnancy loss, with improved predictability compared to ultrasonography and standard histological examination. We propose to use the method of histochemical evaluation of the reticulin–collagen pattern in order to stratify groups of women of fertile age at risk of reproductive failure.

## Figures and Tables

**Table 1:** Demographic and baseline clinical characteristics.

Patient Characteristics	Groups (N-100)		*p*-Value
	Physiological Reproductive Pattern (n-50)	Recurrent Reproductive Failure (n-50)	
Age, mean ± SD	31.14 ± 4.66	32.08 ± 4.37	0.236
Body mass index (BMI), (kg/m^2^)	24.4 (95% CI:23.5–25.3)	24.9 (95% CI:24.0–25.7)	0.389
Pregnancies: number and outcomes			
Gravida, median (IQR)	2 (1)	3 (1)	**0.002**
Clinical pregnancy loss, median (IQR)	0 (0)	2 (1)	**-**
Biochemical pregnancy loss, median (IQR)	0 (0)	1 (2)	**-**
Number of live births, median (IQR)	2 (1)	0 (0)	-
Preterm birth, median (IQR)	1 (2)	0 (0)	-
Term delivery, median (IQR)	2 (1)	0 (0)	-
Hormones			
TSH (uU/mL)	2.41 ± 0.86	2.62 ± 0.92	0.211
Basal FSH (IU/L)	7.81 (95% CI:7.2–8.4)	7.49 (95% CI:6.6–8.3)	0.934
Basal LH (IU/L)	4.92 ± 2.24	4.86 ± 1.42	0.667
Basal serum estradiol (E2) (pg/mL)	39.03 ± 17.48	42.40 ± 21.82	0.627

Abbreviations: IQR— Interquartile range; TSH—thyroid-stimulating hormone; FSH—follicle-stimulating hormone; LH—luteinizing hormone.

**Table 2:** Histochemical reticulin–collagen pattern of endometrial extracellular matrix remodeling in the middle stage of the ovarian cycle secretion phase.

Correspondence to Ovarian Cycle	Physiological Reproductive Status		Recurrent Reproductive Failure	
	Yes	No	Yes	No
Normal reticulin–collagen pattern (NP)	46 (92%)	4 (8%)	22 (44%)	28 (56%) *
Natural cycles, n (%)	46 (100%)	4 (100%)	7 (28.6%)	9 (32.1%)
Stimulated cycles, n (%)	-	-	15 (71.4%)	19 (67.9%)
Pathological stromal reticulin pattern without collagenization (aNP)	4 (8%)	46 (92%)	21 (42%) *	29 (58%)
Natural cycles, n (%)	4 (100%)	46 (100%)	6 (28.6%)	10 (34.5%)
Stimulated cycles, n (%)	-	-	15 (71.4%)	19 (65.5%)
Pathological stromal reticulin pattern with collagenization (aNPC)	0 (0%)	50 (100%)	7 (14%) *	43 (86%)
Natural cycles, n (%)	-	50 (100%)	3 (42.9%)	13 (30.2%)
Stimulated cycles, n (%)	-	-	4 (57.1%)	30 (69.8%)

* Statistically significant versus physiological reproductive status (*p* < 0.05).

**Table 3:** Ultrasonographic (US) endometrium pattern in the middle stage of the ovarian cycle secretion phase.

US Pattern Correspondence to Cycle	Physiological Reproductive Status		Recurrent Reproductive Failure	
	Yes	No	Yes	No
Total number, n (%)	40 (80%)	10 (20%)	35 (70%)	15 (30%)
Natural cycles, n (%)	40 (100%)	10 (100%)	10 (28.6%)	6 (40.0%)
Stimulated cycles, n (%)	-	-	25 (71.4%) *	9 (60.0%)

* Statistically significant versus endometrium pattern from normal ovarian cycle (*p* < 0.05).

**Table 4:** Histological pattern of endometrial glandular epithelium in the middle stage of the ovarian cycle secretion phase.

Correspondence to Cycle	Physiological Reproductive Status		Recurrent Reproductive Failure	
	Yes	No	Yes	No
Total number, n (%)	43 (86%)	7 (14%)	38 (76%)	12 (24%)
Natural cycles, n (%)	43 (100%)	7 (100%)	11 (28.9%)	5 (41.7%)
Stimulated cycles, n (%)	-	-	27 (71.1%) *	7 (58.3%)

* Statistically significant versus endometrium pattern for normal ovarian cycle (*p* < 0.05).

**Table 5:** Relative number of pinopodes of the superficial endometrial epithelium in the middle stage of the ovarian cycle secretion phase.

Relative Number of Pinopodes	Physiological Reproductive Status		Recurrent Reproductive Failure	
	>50%	<50%	>50%	<50%
Total number, n (%)	34 (68%)	16(32%)	26(52%)	24(48%)
Natural cycles, n (%)	34 (100%)	16(100%)	9(34.6%)	7(29.2%)
Stimulated cycles, n (%)	-	-	17(65.4%)	17(70.8%)

## Data Availability

The data supporting this study’s findings are available on request from the corresponding author.
